# Evaluating the Efficiency of *Areke* Distillation Process Using the Traditional Method of Distillation and Double Pipe Distillation: A Traditional Ethiopian Beverage

**DOI:** 10.12688/f1000research.163423.2

**Published:** 2026-01-10

**Authors:** Mulugeta Fentahun, Ayinalem Worku

**Affiliations:** 1 Department of Biology, Debre Markos University, Debre Markos, Amhara, 269, Ethiopia; 2 Mechanical Engineering, Aksum University, Aksum, Tigray, 1010, Ethiopia

**Keywords:** Double pipe distillation, Ethanol, Firewood, Cereal crops, Modified stove, Traditional distillation, Sensory evaluation.

## Abstract

**Background:**

*Areke* is a popular traditional distilled beverage in semi-urban and rural areas in Ethiopia. Traditional *areke* distillation uses an open-fire system that consumes a lot of firewood and produces a large amount of indoor air pollution.

**Methods:**

The *areke* distiller apparatus (heat exchanger, condenser, energy-efficient stoves, storage tanks, and local *areke* extraction apparatus) was manufactured by technicians (welders). Different types of grains (wheat, millet, lupine, barley, and maize) were purchased from a neighborhood market. The traditional method of *areke* fermentation was prepared by an experienced woman brewer using a combination of ingredients and appropriate steps and procedures. The efficacy of a traditional stove, modified stove, and a combination of modified stove and double-pipe were evaluated *.* The amount of ethanol was estimated by measuring its refractive index and specific gravity. The sensory evaluation of *areke* samples was conducted by 10 consumer sensory panelists.

**Result:**

The highest ethanol concentration in *areke* (53.75 ± 0.01 (% v/v)) was obtained from millet E ( *dagusa* E) in double-pipe distillation (E). The maize E ( *bekolo* E) of overall acceptance had the greatest score (4.5 ± 0.01) compared to other *areke* sensory parameters. The alcoholic strength of lupine E ( *gibeto* E) was scored as excellent (5.0 ± 0.01) compared to other *areke* sensory parameters. All judges agreed that traditional and double-pipe *areke* consumption was acceptable. The combination of double-pipe distillation and modified stove resulted in a 50% ± 0.15 reduction in the average amount of firewood used. The traditional open fire stove consumed more firewood (5.1 kg ± 0.1) than the combination of double-pipe distillation and modified stove (2.5 kg ± 0.01).

**Conclusion:**

These results indicate that the combination of double-pipe distillation with a modified stove had better performance than traditional *areke* distillation.

## 1. Introduction

*Areke* is a traditional, home-brewed alcoholic beverage widely consumed in Ethiopia. ^1, 2^ Dembecha, Arsi Negele, and Debre Birhan are the most well-known *areke*-producing regions. ^2– 4^ *Areke* is produced by many households and sold in Ethiopian markets as well as exported to nearby countries. ^3^ A household uses 450 kg of firewood on average for the traditional distillation process to produce 150 liters of *areke* within six business days. ^5^ Many Ethiopian women with low incomes who live in rural and semi-urban areas rely on *areke* as their primary source of income. ^1^ *Areke* is a colorless, higher-alcohol distilled beverage, indigenous to Ethiopia. ^6^ *Areke* usually has an alcohol content between 30% and 50% (v/v). ^7^ It is considered a very strong drink and is more expensive than most other local beverages. ^8, 9^

Locally produced fermented *areke* has been created by indigenous peoples utilizing primitive equipment made of gourds and wood and locally accessible raw materials. ^10^ *Areke* is made by distilling a mixture of water, ground *gesho* ( *Rhamnus Prinoides* L.), and cereals. Barley ( *Hordeum vulgare* L.), wheat ( *Triticum sativum* L.), maize ( *Zea mayz* L.), millet ( *Eleusine coracana* L.), sorghum ( *Sorghum bicolor* L.), teff ( *Eragrostis tef* L.), and other grains are used for producing *areke* beverage. ^4, 11^ *Areke* is traditionally divided into two categories, such as *terra-areke* and *dagim-areke.* The word *terra* in Amharic means ordinary, whereas *dagim* means second time, indicating that it has undergone a second distillation. The normal alcohol content of *dagim-areke* is approximately 45%. The alcoholic content of *terra-areke* was reported to be 34.09% (v/v). ^12, 13^

The distillation process *areke* was described according to Refs. 14 and 15. *Areke* distillation stoves are traditionally made of mud with a round-shaped design, and at least three stones are needed for the pot to stand. The distillation process included a clay pot, pot lid, condensation tube, and collecting flask. A small clay pot ( *madiga*) was used to boil the fermented mash to release the vapor. The cloth was used to seal the *madiga* to prevent vapor loss. A hollow tube of dry bamboo ( *Bambusa vulgaris* L.) was used as a pipe to transport the vapor of *areke* from a clay pot (boiler) to the collector. *Koda* was used as a collector. *Tofaa* made of clay contained cooling water in which the *koda* vapor changed into liquid. ^3, 14^ Local *areke* distillation uses an open-fire system in which much of the heat is wasted on the environment. This forces them to use more firewood, and the energy utilization system is not economical. ^3, 16^ An improved *areke* stove is a stove that requires less firewood to distill the *areke* amount than a traditional one. ^15^ It also produces less smoke compared to traditional stoves. ^16– 18^ Improved *areke* stoves significantly reduce the smoke by having excess air and better combustion. ^15, 19^

No extensive research has been conducted in Ethiopia to enhance the conventional *areke* distillation process using efficient modified *areke* distillers or stoves. Thus, there is a strong motive for increasing the distillation process performance by developing double-pipe heat exchangers and energy-efficient distillation stoves, which lower the production time, increase the amount of *areke* produced, and minimize the utilization of energy. However, traditional fermentation processes and distillation have the potential to transform home-based arts into modern industry necessities through research and technological modification and/or development. Traditional *areke* distillation could be increased by developing a modified fermenter, modified stove, and modified distiller to increase the taste, aroma, content of alcohols and shorten the processing time of the beverage. ^2, 10^

## 2. Methods

### 2.1 The study area

The study was conducted at the Debre Markos microbiology laboratory of the Department of Biology and Mechanical Engineering Department Workshop at Debre Markos University, Ethiopia. The university is located in the town of Debre Markos, which is situated at a latitude and longitude of 10020′N 37043′E/10.3300N 37.7170E and 2,446 meters above sea level. Debre Markos is found 265 kilometers from Bahir Dar and 300 kilometers from Addis Ababa, the capital of Ethiopia. In Debre Markos, 107,684 residents comprise 49,893 men and 57,791 women. ^20^ The lowest and highest temperatures are 15°C and 22°C, respectively, and the average annual rainfall is 380 mm.

### 2.2 *Areke* distillation equipment

**Figure 1.**
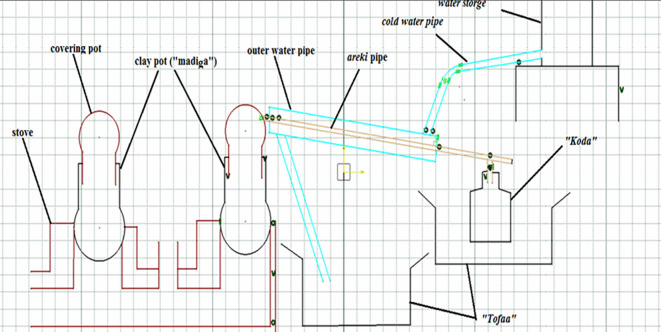
The new double pipe distillation of *areke* model.

The initial steps in improving the efficiency of traditional *areke* fermentation distillation included developing a prototype of the *areke* distiller equipment and gathering various engineering materials ( Figure 1). The equipment required for developing a prototype of the *areke* distiller included a heat exchanger, condenser, energy-saving stoves, fermentation equipment, storage tanks, and local *areke* extraction apparatus. *Areke* distiller equipment was purchased, and the prototype of the *areke* distiller apparatus was manufactured by technicians (welders).

### 2.3 Manufacturing double pipe heat exchanger

A double-pipe heat exchanger (surface condenser) was used to condense the ethanol vapor. The outer pipe was constructed using polyvinyl chloride with a length of 120 cm and a diameter of 3.81 cm ( Figure 3). A large volume of cold water was effectively transported to the outside pipe because the substance was inert and could not react with other materials to cool. The internal pipe was made from copper and stainless steel pipes with a length of 140 cm and a diameter of 1.27 cm. The outer pipe and holes between the outer and inner pipes were sealed using a sealing material.

### 2.4 Stove Construction for *areke* production

A modified *areke* stove and a traditional stove were the two biomass stoves used in the study. Energy-efficient modified *areke* stoves were constructed and evaluated at the *areke* vendor houses as alternative stove types with six stoves in a row, four stoves in a row, two stoves in a row, and one stove. The traditional *areke* stove is constructed from mud and painted with wet manure. A modified fire stove ( *midija*) was constructed using bricks and mud. Smaller granules of crushed clay were sieved through a 4 mm sieve to obtain fine granules. Sifted sawdust and clay in a ratio of 1:1 were mixed by adding water to make them moldable. A closed fire stove ( *midija*) was used to save heat from going out. The bowl-shaped cover was made of clay to fit well with the clay pot and double pipe.

### 2.5 Substrates used in local *areke* production

Five different types of cereal crops were utilized to produce local *areke*, including millet ( *Pennisetum glaucum* L.), wheat ( *Triticum aestivum* L.), lupine ( *white lupine* L.), barley ( *Hordeum vulgare* L.), and maize ( *Zea mayz* L.). *Areke* was made through distillation from various ratios of water, ground *gesho* ( *Rhamnus prinoides* L.), and cereals. ^4, 11, 21^ Various grains (maize, barley, lupine, wheat, and millet) were purchased from a neighborhood market.

### 2.6 Traditional *areke* fermentation process

**Figure 2.**
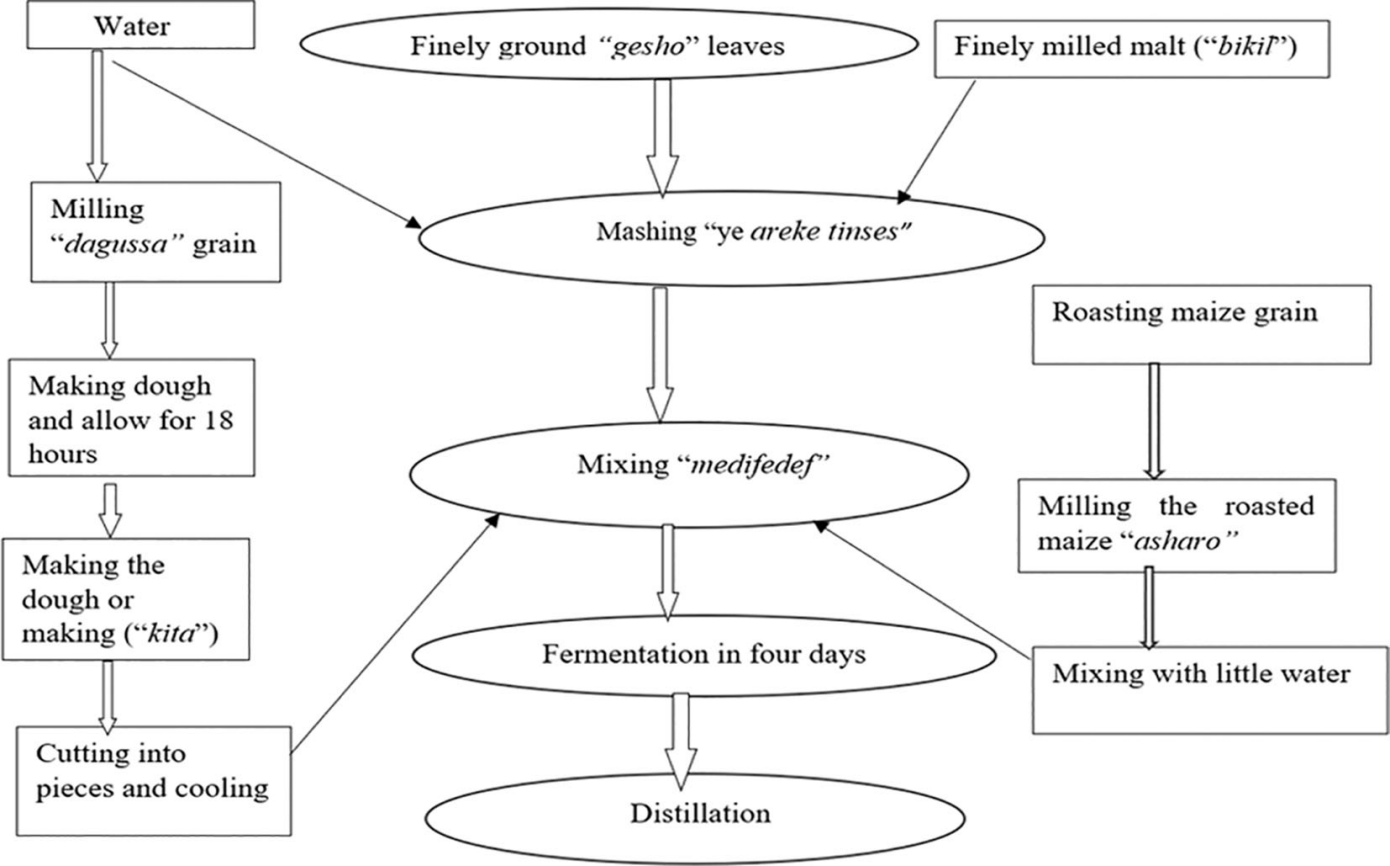
Local *areke* fermentation process.

*Yereke-tensis *, *medifedef*, and *areke* are the three basic processes in the *areke* production process ( Figure 2). ^4, 7, 21, 22^ The traditional *areke* fermentation process was prepared by an experienced woman brewer using a mixture of ingredients with appropriate steps and procedures in Debre Markos City at Bole Kebele, which is held by a private *areke* producer with cash. *Gesho* powder (1 kg), *bikil* malt (0.5 kg), and 12 liters of water were mixed in a fermenter to produce *yereki-tensis,* which was fermented for a week. Cereals were soaked, dried, roasted, and ground to make *kita* (thin pancake-like bread) and *enkuro* (toasted flour). While *enkuro* was prepared by steaming roasted maize flour on a *bret mitad* at 70–100°C, *kita* was cooked on a *mitad*. To create *medifedef*, 10 kg of *enkuro* and 5 kg of *kita* were added to *yereki-tensis* and fermented anaerobically for 15–20 days. The fermented mash was boiled for the *areke* distillation process once fermentation was complete. Traditional *areke* fermentation processes were based on natural, spontaneous fermentation without the intentional inoculation of commercial yeast.

### 2.7 Components of double pipe *areke* distillation

Big pots ( *metensesha*) from the local alcohol distiller were used as fermenters. A small clay pot ( *madiga*) was used to boil the fermented mash to release vapor. A smaller clay pot ( *madiga*) was used during distillation to boil the fermented mash and release vapor, typically holding 12 L depending on local production methods. Hollow tubes of copper and stainless steel were used as pipes to transport the vapor of *areke* from the clay pot (boiler) to the collector. *Koda* was used as a collector. The tubes were made of metal or aluminum to collect vapor that passed through them. *Tofaa* made of clay was used to contain cooling water, in which *koda* vapor changed into a liquid. A traditional distillation apparatus of the bamboo tube (a pipe to transport vapor that comes from the boiler to the collector) made from hollow plant steam ( *Bambusa vulgaris* L.) was used as a control according to the method of distillation described. ^3, 14^

### 2.8 Evaluation of stove and double pipe efficiency

This study was conducted to evaluate the efficacy of a traditional stove, a modified stove, and a combination of a modified stove and double pipe in terms of firewood consumed, time of operation reduced, and productivity of alcohol. The quantity of fuelwood used at a household level was estimated by measuring fuelwood in kilograms (kg) using a spring balance. The calculation of efficiency firewood, time of operation is reduced, and productivity of ethanol amount were calculated according to the Refs. 23, 24 and 25 equation, respectively.

$$\text{Productivity of ethanol amount}=\frac{\text{Amount produced}\times \text{ethanol content}\;(V/V)\times \text{Density of ethanol}}{\text{Time of production}}$$ (1)

$$\text{Time of operation is reduced}\;\mathrm{by}=\frac{\text{Time taken for traditional}-\text{Time taken for double pipe}}{\text{Time taken for traditional}}\times 100$$ (2)

$$\text{Efficiency firewood}=\frac{\text{Firewood consumed for traditional}-\text{Double pipe}}{\text{Firewood consumed for traditional}}\times 100$$ (3)

### 2.9 Determination of ethanol level

The specific gravity and refractive index were measured to estimate the ethanol level using the technique of. ^26^ The specific gravity and refractive index of the samples were determined at 20°C. Ethanol concentration samples were determined using a hydrometer (to measure specific gravity) and a refractometer (to record sugar content). A refractometer (Model 2WA) was used to measure the refractive index of the *areke* samples. A single drop of ethyl alcohol was used for calibration after the prism was cleaned with ethyl alcohol. The apparatus was set up such that half of the field was illuminated and the other half was left in the dark. The refractive index was determined by placing a drop of *areke* on the sample holder. The ethanol content (%) was calculated according to the equation below: Ethanol content = R − [(S.G.−l) × 1000] where R = refractometer reading, SG = specific gravity.

### 2.10 Sensory evaluation of *areke*

The sensory evaluation of *areke* samples was performed by ten consumer sensory panelists. The average age of the panelists was between 25 and 50 years, all of whom had prior experience consuming traditional *areke*. Additionally, the panelists were chosen from the community based on their familiarity with the product, availability, and interest. The ten consumer sensory panelists received formal sensory training prior to the evaluation, which included practice sessions using the hedonic rating scale to ensure consistent and trustworthy assessments, explanations of the sensory qualities of *areke,* and familiarization with the evaluation procedures. Sensory parameters such as flavor, color, alcoholic strength, and overall acceptance of *areke* were evaluated using a hedonic scale of 1 to 5, where excellent = 5, very good = 4, good = 3, Fair = 2, and poor = 1. Sensory attributes (color, taste, alcoholic strength, and overall acceptability) were assessed utilizing the technique of. ^27– 29^

### 2.11 Data analysis

The SPSS version 23.0 IBM SPSSInc., Chicago, IL, SPSS (RRID: SCR_002865) ( https://www.ibm.com/support/pages/downloading-ibm-spss-statistics-23) was used to evaluate the data. The averages and standard deviations of the triplicates analysis were determined using analysis of variance (ANOVA). Tukey’s multiple range testing was defined as the statistical significance (p < 0.05).

### 2.12 Ethical consideration

The present study was approved by the Ethical Review Committee of Debre Markos University, College of Natural and Computational Sciences (Ref No. DU/NCS/12/2024) on the date of approval, December 10, 2024. This study adhered to the ethical principles outlined in the Declaration of Helsinki. Participants verbally consented to promote a more conversational dialogue than in a written form. The participants’ verbal consent was approved by the ethics committee. The ethical approval committee waived the participant consent because the study involved minimal risk to participants and did not adversely affect their rights and their potential benefits to society. Participants verbally consented on May 7, 2024, for their data to be used. All data were de-identified using the Safe Harbor method to ensure the protection of personal and sensitive information.

## 3. Results and Discussion

### 3.1 Physicochemical properties of *areke*

The physicochemical properties of *areke* obtained from traditional distillation (C) and double-pipe distillation (E) are shown in ( Table 1). There was no statistically significant (p ≥ 0.05) difference between barley E *(gebis* E) and wheat E ( *sinda* E) in the ethanol content of *areke* samples. The study indicated that double-pipe distillation (E) produced a higher ethanol concentration than traditional distillation (C). The highest concentration of ethanol in *areke* was achieved through double pipe distillation (E) from millet E ( *dagusa* E) 53.75 ± 0.01 (% v/v). The minimum ethanol concentration of *areke* was achieved through traditional distillation (C) from lupine C ( *gibeto* C) 24.19 ± 0.05 (% v/v). Medium levels of ethanol were found in five of the *areke* samples of millet E ( *dagusa* C), maize E ( *bekolo* E), barley C ( *gebis* C), and lupine C ( *gibeto* C). The double pipe and traditional distillers *areke* ethanol contents were consistent with those reported in other studies, ^7, 12, 30^ which implies that *areke* ethanol content can vary greatly. The differences in the ethanol content of *areke* may be due to variations in cereal crops, methods of preparation, fermentation, and modified distillers. ^31– 34^

**Table 1.** Physicochemical properties of double pipe distillation (E) and traditional distillation (C) *areke* samples.

*Areke* types	Ethanol content	Specific gravity	Refractive index
Lupine E ( *Gibeto* E)	53.61 ± 0.11 ^a^	0.93 ± 0.01 ^a^	1.36 ± 0.05 ^a^
Lupine C ( *Gibeto* C)	24.19 ± 0.05 ^c^	0.97 ± 0.00 ^d^	1.33 ± 0.00 ^bd^
Barley E ( *Gebis* E)	42.79 ± 0.04 ^b^	0.95 ± 0.00 ^b^	1.35 ±0.00 ^ad^
Barley C ( *Gebis* C)	37.81 ± 0.05 ^d^	0.99 ± 0.00 ^e^	1.42 ± 0.01 ^c^
Wheat E ( *Sinda* E)	42.80 ± 0.01 ^b^	0.95 ± 0.00 ^bc^	1.34 ± 0.01 ^ad^
Wheat C ( *Sinda* C)	40.39 ± 0.03 ^e^	0.93 ± 0.01 ^a^	1.36 ± 0.00 ^a^
Millet E ( *Dagusa* E)	53.75 ± 0.01 ^f^	0.92 ± 0.01 ^f^	1.36 ± 0.01 ^a^
Millet C ( *Dagusa* C)	31.56 ± 0.01 ^g^	0.96 ± 0.02 ^h^	1.34 ± 0.10 ^d^
Maize E ( *Bekolo* E)	38.78 ± 0.01 ^h^	0.96 ± 0.02 ^c^	1.35 ± 0.01 ^ad^
Maize C ( *Bekolo* C)	35.92 ± 0.02 ^i^	0.94 ± 0.00 ^i^	1.35 ± 0.00 ^ad^

E = Double pipe *areke* distillation; C = Tradational *areke* distillation; Values are mean ± standard deviation of three replicates; Means within a row with different superscripts differ significantly (p ≤ 0.05).

The average specific gravity of traditional distillation *areke* ranged from 0.99 ± 0.00 to 0.92 ± 0.01 ( Table 1). Barley C ( *gebis* C) and lupine C ( *gibeto* C) exhibited higher specific gravity than the other *areke* samples. The double pipe distillation *areke* had a mean specific gravity that varied between 0.96 ± 0.02 and 0.92 ± 0.01. The average refractive indices of the *areke* samples varied between 1.42 ± 0.01 and 1.33 ± 0.00. A higher refractive index was obtained from barley C ( *gebis* C) than from other *areke* samples. The mean specific gravity and refractive index findings in this study were consistent with those of other researchers. ^25, 35– 37^ This difference is due to variations in fermentation and distillation efficiency levels.

### 3.2 Sensory evaluation of *areke*

The sensory test (taste, color, alcoholic strength, and general acceptance) of *areke* was conducted by a panel of ten people, and the average results are presented in Table 2. Lupine C ( *gibeto* C) had the lowest score (2.2 ± 0.01) among the measures for overall acceptance compared to the other parameters. The highest score was attributed to maize E ( *bekolo* E) (4.5 ± 0.01) for overall acceptance than other *areke* sensory parameters. The alcoholic strength of lupine E ( *gibeto* E) was scored excellent (5.0 ± 0.01) to other *areke* sensory parameters. The taste of maize E ( *bekolo* E) (5.0 ± 0.01) and wheat E ( *sinda* E) (5.0 ± 0.02) were scored excellent based on the sensory assessment. There was a statistically significant (p < 0.05) difference between lupine E ( *gibeto* E), lupine C ( *gibeto* C), and maize E ( *bekolo* E) in the overall acceptance of the *areke* samples. The overall acceptance of double-pipe distillation of *areke* samples was higher than that of traditional distillation. All judges agreed that traditional and double pipe *areke* consumption was acceptable based on their sensory evaluations. The sensory evaluation of *areke* is strongly affected by the method of preparation and the use of *gesho*, which in turn plays an important role in the perception of the people. ^2, 12, 38, 39^ The heat provided during the boiling stage has a significant impact on the final distilled *areke* flavor, aroma, color, and alcoholic strength. ^25^

**Table 2.** Sensory evaluation of traditional and pipe distillation of *areke* samples (Mean ± SD) (n=10).

	Color	Taste	Alcoholic strength	Overall acceptance
Lupine E ( *Gibeto* E)	2.2 ± 0.02 ^a^	1.5 ± 0.02 ^a^	5.0 ± 0.01 ^a^	2.9 ± 0.01 ^a^
Lupine C ( *Gibeto* C)	2.2 ± 0.01 ^a^	1.2 ± 0.01 ^b^	4.5 ± 0.01 ^b^	2.2 ± 0.01 ^b^
Barley E ( *Gebis* E)	4.0 ± 0.02 ^b^	4.5 ± 0.01 ^c^	2.0 ± 0.01 ^c^	3.5 ± 0.01 ^cg^
Barley C ( *Gebis* C)	3.8 ± 0.06 ^c^	4.1 ± 0.01 ^d^	1.8 ± 0.01 ^d^	3.4 ± 0.00 ^c^
Wheat E ( *Sinda* E)	3.5 ± 0.02 ^d^	5.0 ± 0.02 ^e^	3.0 ± 0.02 ^e^	3.8 ± 0.01 ^d^
Wheat C ( *Sinda* C)	3.4 ± 0.02 ^e^	4.7 ± 0.02 ^f^	2.9 ± 0.01 ^f^	3.7 ± 0.00 ^g^
Millet E ( *Dagusa* E)	3.3 ± 0.02 ^f^	3.7 ± 0.01 ^g^	4.0 ± 0.01 ^g^	3.5 ± 0.29 ^cg^
Millet E ( *Dagusa* C)	3.2 ± 0.01 ^g^	3.3 ± 0.01 ^h^	4.0 ± 0.02 ^h^	3.3 ± 0.02 ^cg^
Maize E ( *Bekolo* E)	5.0 ± 0.03 ^h^	5.0 ± 0.01 ^e^	3.4 ± 0.00 ^i^	4.5 ± 0.01 ^e^
Maize E ( *Bekolo* C)	5.0 ± 0.02 ^h^	4.4 ± 0.01 ^i^	3.0 ± 0.01 ^e^	4.0 ± 0.02 ^df^

E = Double pipe *areke* distillation; C = Tradational *areke* distillation; Values are mean ± standard deviation of three replicates; Means within a row with different superscripts differ significantly (p ≤ 0.05).

### 3.3 Evaluation of stove and double pipe efficiency

**Figure 3.**
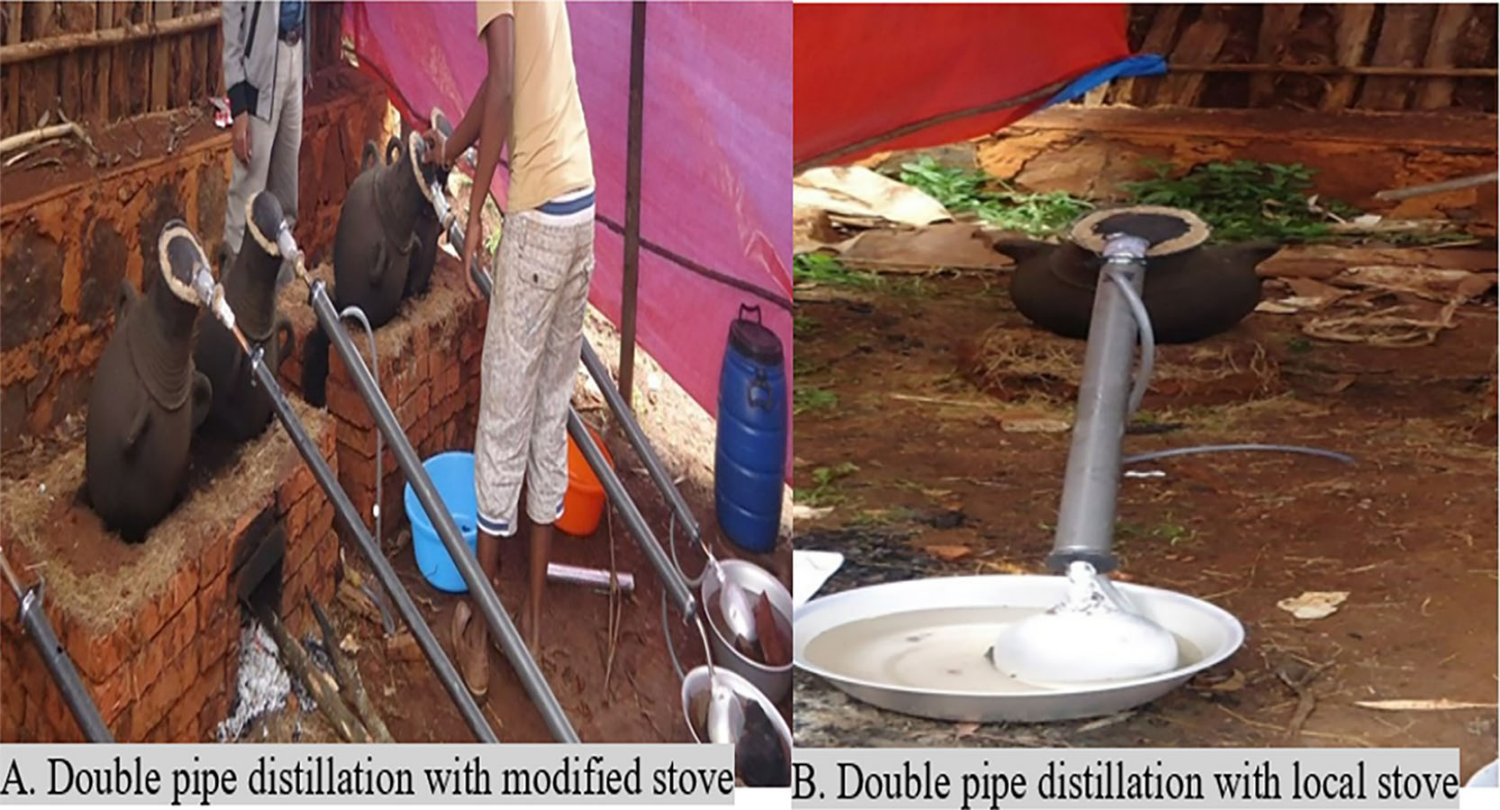
The double pipe distillation with a modified stove and a local stove.

The average amount of firewood used was reduced by 50% **±** 0.15 when double-pipe distillation was combined with a modified stove ( Table 3; Figure 3). The percentage of *areke* (v/v) increased by 68.5% ± 0.01 when the combination of a modified stove and double-pipe distillation were used. The amount of *areke* produced from traditional distillation was 1013 ml ± 1.00, and the temperature of the *areke* was lowered to 61°C ± 1.00 as soon as the distillation stopped ( Table 3). The amount of *areke* in the modified stove distillation was increased by 1014 ml ± 0.57, and the temperature of the *areke* was lowered to 60°C ± 0.57 as soon as the distillation stopped. The amount of *areke* in double-pipe distillation was increased by 1141ml ± 1.00, and the temperature of *areke was* reduced by 35°C ± 1.15 as soon as distillation stopped. The amount of *areke* in a modified stove and double pipe distillation increased by 1253 ml ± 0.92, and the temperature of the *areke* dropped by 35°C ± 0.58 as soon as the distillation stopped. Double-pipe distillation was more efficient than traditional bamboo *areke* distillation because of improved condensation and heat transfer. The alcohol vapor traveled through an inner pipe and was continuously condensed into a liquid by cooling water in the outer pipe of the double-pipe distillation.

**Table 3.** The efficiency of traditional, double pipe, modified stove, and double pipe distillation with modified stove.

Parameters	Traditional distillation	Double pipe distillation	Modified stove distillation	Combination of modified stove and double pipe distillation
Time for distillation	2.46 hrs ± 0.01 ^a^	2.32 hrs ± 0.01 ^b^	2.32 hrs ± 0.00 ^b^	1.31 hrs ± 0.01 ^c^
Amount of *areke* produced	1013 ml ± 1.00 ^a^	1141ml ± 1.00 ^b^	1014 ml ± 0.57 ^a^	1253 ml ± 0.92 ^c^
The temperature of *areke* at the of end distillation	61°C ± 1.00 ^a^	35°C ± 1.15 ^b^	60°C ± 0.57 ^a^	35°C ± 0.58 ^b^
Firewood consumed	5.1 kg **±** 0.1 ^a^	3.6 kg **±** 0.01 ^b^	3.5 kg **±** 0.01 ^b^	2.5 kg **±** 0.01 ^c^
Percentage of *areke* (v/v)	40.4% ± 0.01 ^a^	45.4% ± 0.01 ^b^	42.4% ± 0.01 ^c^	68.5% ± 0.01 ^d^
Time of operation reduced	_	5.4% ± 0.01 ^a^	7.6% ± 0.01 ^b^	42% ± 0.58 ^c^
Productivity of *areke*	2.0 g/min ± 0.05 ^a^	2.5 g/min ± 0.01 ^b^	2.1 g/min ± 0.05 ^a^	7.3 g/min ± 0.06 ^c^
Firewood saved	_	28% **±** 0.17 ^a^	30% **±** 0.12 ^b^	50% **±** 0.15 ^c^

Values are mean ± standard deviation of three replicates; Means within a row with different superscripts differ significantly (p ≤ 0.05).

Firewood consumption was higher in the traditional open fire stove (5.1 kg **±** 0.1) than in the modified stove (3.5 kg **±** 0.01). A total of 2.5 kg **±** 0.01 of firewood was needed for distillation using a combination of a modified stove and double-pipe distillation ( Table 3; Figure 3). Traditional and modified stove distillation methods were not statistically significant (p > 0.05) in terms of *areke* productivity, temperature at the end of distillation, or volume of *areke* produced. According to Temesgen and Kamil, ^15^ the time used by the modified ( *mirt) areke* stove was reduced by over 50% by the stove with three stones (52%; 1:04 h: min). The modified *areke* stove reduced distilling time by 22% compared to the traditional *areke* stove. ^14^ The modified *areke* stove emits less smoke and uses less firewood to distill *areke* than a traditional stove. ^15– 17, 40^ Combining the modified stove with double-pipe distillation resulted in a productivity of 7.3 ± 0.06 g/min, which is more than three times higher than that of the conventional method (2.0 ± 0.05 g/min). This is due to reduced heat loss, increased vapor generation, and accelerated alcohol recovery. ^41^

## 4. Conclusion

According to the study, the ethanol concentration from double-pipe distillation (E) was higher than that from traditional distillation (C). The maximum ethanol concentration of *areke* was obtained from millet E ( *dagusa* E) (53.75 ± 0.01 (% v/v)) and Lupine E ( *gibeto* E) (53.61 ± 0.11). The maximum score attributed was obtained from maize E ( *bekolo* E) (4.5 ± 0.01) and maize E ( *bekolo* C) (4.0 ± 0.02) parameters of overall acceptance than other *areke* sensory parameters. Our findings show a better reduction in time (42% ± 0.58) and amount of firewood (50% **±** 0.15) to distill *areke* using a combination of double-pipe distillation with a modified stove. The performance of traditional *areke* distillation can be scaled up by developing a modified distiller (double-pipe distillation) and a modified stove.

## Ethical approval and consent

Ethical approval was obtained from the Debre Markos University, College of Natural and Computational Sciences of Ethical Review Committee approval number (Ref No. DU/NCS/12/2024) on the date of approval,10 ^th^ December 2024. The ethical guidelines of the Declaration of Helsinki were followed in this investigation. Participants verbally consented to facilitate more natural conversations than written forms. Participants verbally consent were approved by the ethical committee. Participants were provided with detailed information about the study objectives and benefits before they provided verbal consent. The ethical approval committee decided to waive participant consent because the study involved minimal risk to participants and did not adversely affect their rights and their potential benefits to society.

## Data Availability

Figshare: SPSS Data: Physicochemical properties of double pipe distillation (E) and traditional distillation (C) *areke* samples. https://doi.org/10.6084/m9.figshare.28639451. ^42^ This project contains the following underlying data:
- SPSS Data: Sensory evaluation of traditional and pipe distillation of *areke* samples - SPSS Data: The efficiency of traditional, double pipe, modified stove, and combination of modified stove and double pipe distillation -
Figure 1 S: Traditional *areke* distillation -
Figure 2 S: Modified stove firing -
Figure 3 S: Traditional distillation of local *areke*
-
Figure 4 S: Combination of double pipe distillation with traditional stove -
Figure 5 S: Combination of double pipe distillation with modified stove Data are available under the terms of the Creative Commons Zero “No rights reserved” data waiver (CC0 1.0 Public domain dedication).
