# Supplementary material for: Evaluating the Efficiency of Areke Distillation Process Using the Traditional Method of Distillation and Double Pipe Distillation: A Traditional Ethiopian Beverage
Source: F1000Res. 2026 Jan 10;14:682. Originally published 2025 Jul 10. [Version 2] doi: 10.12688/f1000research.163423.2 (PMC12820471; doi:10.12688/f1000research.163423.2)
Supplement: Peer review history

# Reviewer report: Reviewer response for version 2

**DOI:** 10.5256/f1000research.193584.r449116

**Published:** 2026-01-20

**Authors:** Nermina Spaho

**Affiliations:** 1 University of Sarajevo, Sarajevo, Bosnia and Herzegovina

After the accepted corrections, I believe the manuscript is now ready for indexing.

Is the work clearly and accurately presented and does it cite the current literature?

Partly

If applicable, is the statistical analysis and its interpretation appropriate?

Yes

Are all the source data underlying the results available to ensure full reproducibility?

Yes

Is the study design appropriate and is the work technically sound?

Partly

Are the conclusions drawn adequately supported by the results?

Yes

Are sufficient details of methods and analysis provided to allow replication by others?

Partly

Reviewer Expertise:

Food technology: distillation, aroma compounds

I confirm that I have read this submission and believe that I have an appropriate level of expertise to confirm that it is of an acceptable scientific standard.

# Reviewer report: Reviewer response for version 2

**DOI:** 10.5256/f1000research.193584.r449117

**Published:** 2026-01-12

**Authors:** Diego Bonatto

**Affiliations:** 1 Universidade Federal do Rio Grande do Sul, Porto Alegre, Brazil

The revised manuscript can be accepted for indexing.

Is the work clearly and accurately presented and does it cite the current literature?

Yes

If applicable, is the statistical analysis and its interpretation appropriate?

Yes

Are all the source data underlying the results available to ensure full reproducibility?

Partly

Is the study design appropriate and is the work technically sound?

Partly

Are the conclusions drawn adequately supported by the results?

Yes

Are sufficient details of methods and analysis provided to allow replication by others?

Partly

Reviewer Expertise:

Yeast Genetics and Molecular Biology; Yeast Fermentation.

I confirm that I have read this submission and believe that I have an appropriate level of expertise to confirm that it is of an acceptable scientific standard.

# Reviewer report: Reviewer response for version 1

**DOI:** 10.5256/f1000research.179774.r406810

**Published:** 2025-09-15

**Authors:** Diego Bonatto

**Affiliations:** 1 Universidade Federal do Rio Grande do Sul, Porto Alegre, Brazil

The manuscript provides a valuable contribution to documenting and improving traditional distillation practices of *areke*, a culturally and economically crucial Ethiopian beverage. The comparison between the traditional distillation system and a double-pipe model combined with modified stoves is of applied interest, with potential benefits in sustainability, reduction of firewood use, and minimization of indoor air pollution. Highlighting the direct impact on fuel consumption and alcohol yield underscores the social and environmental relevance of the study.

 Another positive aspect is the inclusion of physicochemical analyses (refractive index, specific gravity, ethanol content) along with sensory evaluations conducted by local panelists, which provides an integrated view of the final product quality. Data collection was performed in triplicate and analyzed statistically (ANOVA with Tukey’s test), adding robustness to the conclusions.

 Furthermore, the article presents original field data and demonstrates ethical responsibility in conducting the study, including institutional approval and informed consent from participants. The availability of underlying data in a public repository (Figshare) complies with open science practices, ensuring transparency and partial reproducibility of the results.

*Major Points:*

1. The absence of photographs or clear illustrations of the distillation apparatus (traditional, modified stove, and double-pipe system) limits the clarity of the methodology and hinders replicability.
2. Methodological details are insufficient: exact amounts of raw materials, whether the cereals were ground and how, and the volume of distillation vessels are missing.
3. The fermentation process is described too superficially, with no details on the microorganisms (yeasts, bacteria), active enzymes, or fermentation times, which undermines the scientific basis of the comparison.
4. The sensory evaluation has limitations: small panel size (n=10), unclear recruitment criteria, and no description of panelist training. These factors reduce the reliability and generalizability of the results.
5. Some calculations of energy efficiency and productivity are not fully contextualized or compared with international literature. A critical discussion on how these values align (or diverge) from global distillation standards is lacking.
6. The article is primarily descriptive, with limited mechanistic discussion on why the double-pipe system provides higher efficiency.
7. Terminology inconsistencies occur throughout the text (e.g., “flavor” vs. “taste”), which may confuse readers.

*Minor Points:*

1. In section 2.9 (Determination of ethanol level), the phrase “SG = SG = specific gravity” contains a redundant “SG =” that should be deleted.
2. In section 2.10 (Sensory evaluation), “flavor” should be replaced with “taste,” as the actual sensory parameter evaluated was taste, not aroma.
3. Typographical error: “Zea mayz L.” should be corrected to “Zea mays L.”
4. Spacing/typographical issues appear, e.g., “arekewas” or “waswa.”
5. The English writing requires refinement, particularly in terms of sentence structure, as evidenced by overly long and repetitive sentences (e.g., “ethanol content (v/v) of areke ranges from 30% to 50%. This drink is priced higher than other local beverages and is frequently considered highly powerful to drink.”). Professional language editing is recommended.
6. Some table formatting regarding statistical significance superscripts is unclear and may be difficult for readers unfamiliar with the notation.
7. References are inconsistently formatted: some include “Publisher Full Text” while others do not.

Is the work clearly and accurately presented and does it cite the current literature?

Yes

If applicable, is the statistical analysis and its interpretation appropriate?

Yes

Are all the source data underlying the results available to ensure full reproducibility?

Partly

Is the study design appropriate and is the work technically sound?

Partly

Are the conclusions drawn adequately supported by the results?

Yes

Are sufficient details of methods and analysis provided to allow replication by others?

Partly

Reviewer Expertise:

Yeast Genetics and Molecular Biology; Yeast Fermentation.

I confirm that I have read this submission and believe that I have an appropriate level of expertise to confirm that it is of an acceptable scientific standard, however I have significant reservations, as outlined above.

# Response

**Published:** 2025-12-13

**Authors:** Mulugeta Fentahun

**Affiliations:** Department of Biology, Debre Markos University, Debre Markos, Amhara, Ethiopia

**Mulugeta Fentahun**

 Professor Diego Bonatto thank you very much for your insightful comments and the time you dedicated to review the manuscript.

 We greatly appreciate your feedback and suggestions. We provide a detailed response to each comment, along with the corresponding changes made in the revised manuscript below:

- **Comment**: The manuscript makes a useful contribution by documenting and improving traditional areke distillation. Comparing the traditional system with the double-pipe model and modified stoves shows practical benefits, including reduced fuel use, higher alcohol yield, and lower indoor air pollution. The inclusion of physicochemical measurements and sensory evaluation provides a comprehensive assessment of product quality, and the use of triplicate data with ANOVA adds statistical strength. The study is based on original field data, follows ethical protocols, and shares its dataset on Figshare, supporting transparency and reproducibility.
- **Response**: We appreciate the reviewer’s positive comments on our manuscript.

**Major points:**

- **Comment 1**: The absence of photographs or clear illustrations of the distillation apparatus (traditional, modified stove, and double-pipe system) limits the clarity of the methodology and hinders replicability.

- **Response**: Thank you for this valuable comment. We have now added clear photographs or clear illustrations of the distillation apparatus. These visual materials enhance the clarity of the methodology and improve the replicability of the study (see the revised manuscript).
- **Comment 2**: Methodological details are insufficient: exact amounts of raw materials, whether the cereals were ground and how, and the volume of distillation vessels are missing.
- **Response**: We sincerely thank the reviewer for your constructive feedback. We have now provided detailed methodological information, including the quantities of raw materials, the grinding procedure for the cereals, and the volumes distillation vessels (refer to the revised manuscript).
- **Comment 3**: The fermentation process is described too superficially, with no details on the microorganisms (yeasts, bacteria), active enzymes, or fermentation times, which undermines the scientific basis of the comparison.
- **Response: **Thank you for this comment. We would like to clarify that the areke fermentation in this study relied entirely on natural, spontaneous fermentation without adding commercial yeast or conducting microbial inoculation (see the revised manuscript).
- **Comment 4**: The sensory evaluation has limitations: small panel size (n=10), unclear recruitment criteria, and no description of panelist training. These factors reduce the reliability and generalizability of the results.
- **Response: **Thank you for highlighting these limitations. We have revised this section The sensory panel size (n = 10) was limited due to the availability of experienced local assessors. Participants were recruited voluntarily from experienced areke consumers, and formal panelist training was conducted prior to the evaluation. Please refer to the updated section in the revised manuscript.
- **Comment 5**: Some calculations of energy efficiency and productivity are not fully contextualized or compared with international literature. A critical discussion on how these values align (or diverge) from global distillation standards is lacking.
- **Response: **Thank you for your insightful comment. We have revised the discussion section using the literature, as suggested (see the updated section in the revised manuscript).
- **Comment 6**: The article is primarily descriptive, with limited mechanistic discussion on why the double-pipe system provides higher efficiency.

- **Response: **Thank you for this comment. We have revised the manuscript to include a more mechanistic discussion of the double-pipe system (see the revised manuscript).
- **Comment 7**: Terminology inconsistencies occur throughout the text (e.g., “flavor” vs. “taste”), which may confuse readers.
- **Response: **Thank you for this comment. We have revised the term **“** **flavor”** has been replaced with **“** **taste”** **.**

**Minor Points:**

1. **Comment 1**: In section 2.9 (Determination of ethanol level), the phrase “SG = SG = specific gravity” contains a redundant “SG =” that should be deleted.

- **Response**: Thank you for this comment. The redundant phrase “SG =” in Section 2.9 has been removed.
- **Comment 2**: In section 2.10 (Sensory evaluation), “flavor” should be replaced with “taste,” as the actual sensory parameter evaluated was taste, not aroma.
- **Response**: Thank you for your feedback. In Section 2.10, the term “flavor” has been replaced with “taste”.
- **Comment 3**: Typographical error: “Zea mayz L.” should be corrected to “Zea mays L.”
- **Response: **Thank you for this comment. The typographical error has been corrected; “Zea mayz L.” has been revised to **“** **Zea mays L.”** in the manuscript.
- **Comment 4**: Spacing/typographical issues appear, e.g., “arekewas” or “waswa.”
- **Response: **Thank you for this comment. All such errors (e.g., “arekewas,” “waswa”) have been carefully corrected throughout the manuscript.
- **Comment 5**: The English writing requires refinement, particularly in terms of sentence structure, as evidenced by overly long and repetitive sentences (e.g., “ethanol content (v/v) of areke ranges from 30% to 50%. This drink is priced higher than other local beverages and is frequently considered highly powerful to drink.”). Professional language editing is recommended.
- **Response: **We sincerely thank the reviewer for the valuable suggestion regarding the language quality of our manuscript. We have thoroughly revised the manuscript to improve English clarity, grammar, and readability.
- **Comment 6**: Some table formatting regarding statistical significance superscripts is unclear and may be difficult for readers unfamiliar with the notation **.**
- **Response**: Thank you for this comment. We have revised the manuscript.
- **Comment 7**: References are inconsistently formatted: some include “Publisher Full Text” while others do not.
- **Response: **Thank you for this comment. All references have been revised according to the journal’s required citation style.

Thank you once again for your valuable feedback. Your comments have significantly contributed to improving the quality of this paper.

 Best regards,

 Authors

# Reviewer report: Reviewer response for version 1

**DOI:** 10.5256/f1000research.179774.r399314

**Published:** 2025-09-02

**Authors:** Nermina Spaho

**Affiliations:** 1 University of Sarajevo, Sarajevo, Bosnia and Herzegovina

The manuscript presents the traditional production of arak and the possibilities of improving this production through the adaptation of the distillation apparatus.

 Although the study bears local relevance and does not contribute significantly to the scientific body of knowledge, it provides practical value in the improvement of traditional Areca production practices.

 My main criticism is the lack of photos of the distillation apparatus. Including them would make the described adaptations much clearer, as they are difficult to identify solely from the text.

 The authors cited photo (Figure) from the references that cannot be found online. Therefore, all photos cited by the author should also be shown in the manuscript.

 Furthermore, here are the some other corrections and improvements that should be introduced before publication:

 2.5 Substrates used in local areke production

 The amount of raw material are missed. Are the cereal crops ground and how? Needs a better described.

 2.6 Traditional areke fermentation process

 The fermentation process needs to be describe more precise in sens of yeast and enzyme used.

 2.7 Components of double pipe areke distillation

 The volume of pot is missed

 2.9 Determination of ethanol level

 SG = SG = specific gravity. One SG is surplus, delete it.

 2.10 Sensory evaluation of areke

 Sensory parameters such as flavor, color, alcoholic strength, and overall acceptance of areke were evaluated using a hedonic scale of 1 to 5...

 Flavor should be replaced with taste, because authors are used taste as sensory parameter not flavor.

Is the work clearly and accurately presented and does it cite the current literature?

Partly

If applicable, is the statistical analysis and its interpretation appropriate?

Yes

Are all the source data underlying the results available to ensure full reproducibility?

Yes

Is the study design appropriate and is the work technically sound?

Partly

Are the conclusions drawn adequately supported by the results?

Yes

Are sufficient details of methods and analysis provided to allow replication by others?

Partly

Reviewer Expertise:

Food technology: distillation, aroma compounds

I confirm that I have read this submission and believe that I have an appropriate level of expertise to confirm that it is of an acceptable scientific standard, however I have significant reservations, as outlined above.

# Response

**Published:** 2025-12-13

**Authors:** Mulugeta Fentahun

**Affiliations:** Department of Biology, Debre Markos University, Debre Markos, Amhara, Ethiopia

**Mulugeta Fentahun**

 Thanks to Professor Nermina Spaho for your constructive comments. We greatly appreciate your feedback and suggestions. Please find my responses to your comments below:

- **Comment 1**: My main criticism is the lack of photos of the distillation apparatus. Including them would make the described adaptations much clearer, as they are difficult to identify solely from the text. The authors cited photo (Figure) from the references that cannot be found online. Therefore, all photos cited by the author should also be shown in the manuscript.

- **Response**: Thank you for this comment. We have now added photographs or clear illustrations of the distillation apparatus. These visual materials enhance the clarity of the methodology and improve the replicability of the study (see the revised manuscript).
- **Comment 2**: Substrates used in local areke production section in method. The amount of raw material are missed. Are the cereal crops ground and how? Needs a better described.
- **Response**: We thank the reviewer for your constructive feedback. We have added detailed methodological information, including raw material quantities, cereal grinding procedures, and distillation vessel volumes (see revised manuscript).
- **Comment 3**: Traditional areke fermentation process section in method. The fermentation process needs to be describe more precise in sens of yeast and enzyme used.
- **Response: **Thank you for this comment. We would like to clarify that the areke fermentation in this study relied entirely on natural, spontaneous fermentation without adding commercial yeast or conducting microbial inoculation (see the revised manuscript).
- **Comment 4**: Components of double pipe areke distillation section in method. The volume of pot is missed.
- **Response: **Thank you for this comment. We have added the volume of the pot.
- **Comment 5**: Determination of ethanol level section in method. SG = SG = specific gravity. One SG is surplus, delete it.
- **Response: **Thank you for your comment. We have corrected the typographical error in the “Determination of ethanol level” section. The redundant **“SG =** **”** has been deleted.
- **Comment 6**: Sensory evaluation of the areke section in method. Sensory parameters such as flavor, color, alcoholic strength, and overall acceptance of areke were evaluated using a hedonic scale of 1 to 5. Flavor should be replaced with taste, because authors are used taste as sensory parameter not flavor.
- **Response: **Thank you for the comment. We have revised the sensory evaluation section accordingly. The term **“** **flavor”** has been replaced with **“** **taste”**

Thank you once again for your valuable feedback. Your comments have significantly contributed to improving the quality of this paper.

 Best regards,

 Authors
